# Longitudinal cardiac magnetic resonance imaging following clinical response to rilonacept and prior to recurrence upon treatment suspension: a RHAPSODY subgroup analysis

**DOI:** 10.1093/ehjci/jeae200

**Published:** 2024-08-22

**Authors:** Paul C Cremer, Antonio Brucato, Antonella Insalaco, David Lin, Sushil A Luis, Deborah H Kwon, Christine L Jellis, JoAnn Clair, Allison Curtis, Sheldon Wang, Allan L Klein, Massimo Imazio, John F Paolini

**Affiliations:** Department of Cardiovascular Imaging, Center for the Diagnosis and Treatment of Pericardial Diseases, Cleveland Clinic, Heart and Vascular Institute, Cleveland, OH, USA; Department of Biomedical and Clinical Science, Fatebenefratelli Hospital, University of Milano, Milano, Italy; Division of Rheumatology, IRCCS [European Reference Network (ERN) for Rare Immunodeficiency, Autoinflammatory and Autoimmune Diseases (RITA) Center], Ospedale Pediatrico Bambino Gesù, Rome, Italy; Department of Cardiology, Minneapolis Heart Institute at Abbott Northwestern Hospital, Minneapolis, MN, USA; Department of Cardiovascular Medicine, Mayo Clinic, Rochester, MN, USA; Department of Cardiovascular Imaging, Center for the Diagnosis and Treatment of Pericardial Diseases, Cleveland Clinic, Heart and Vascular Institute, Cleveland, OH, USA; Department of Cardiovascular Imaging, Center for the Diagnosis and Treatment of Pericardial Diseases, Cleveland Clinic, Heart and Vascular Institute, Cleveland, OH, USA; Kiniksa Pharmaceuticals, Lexington, MA, USA; Kiniksa Pharmaceuticals, Lexington, MA, USA; Kiniksa Pharmaceuticals, Lexington, MA, USA; Department of Cardiovascular Imaging, Center for the Diagnosis and Treatment of Pericardial Diseases, Cleveland Clinic, Heart and Vascular Institute, Cleveland, OH, USA; Department of Medicine (DMED), Cardiothoracic Department, University Hospital “Santa Maria della Misericordia”, ASUFC, Udine, Italy; Kiniksa Pharmaceuticals, Lexington, MA, USA

**Keywords:** rilonacept, pericarditis, magnetic resonance imaging, gadolinium, interleukin-1, clinical decision-making

## Abstract

**Aims:**

In the Phase 3 trial, RHAPSODY, rilonacept effectively resolved active pericarditis recurrences, and long-term treatment led to sustained pericarditis recurrence risk reduction. Prior analysis suggested association between higher late gadolinium enhancement (LGE) at baseline and more rapid recurrence upon rilonacept suspension after 12 weeks of treatment. This subgroup analysis assessed the utility of longitudinal serial cardiac magnetic resonance (CMR) imaging for tracking clinical improvement and predicting post-treatment cessation outcomes to help guide clinical decision-making.

**Methods and results:**

At an 18-month decision milestone (18MDM) in the RHAPSODY long-term extension, investigators decided if patients would continue rilonacept, suspend rilonacept for off-treatment observation, or discontinue the study. Pericardial thickness, pericardial oedema (T2-short tau inversion recovery, T2-STIR), and LGE were determined at baseline and 18MDM by an imaging core lab blinded to clinical data, and pericarditis recurrence was investigator-assessed. CMR results in patients with data at both baseline and 18MDM (*n* = 13) showed that pericardial thickness, T2-STIR, and LGE were reduced during rilonacept treatment. Among patients with CMR data who suspended rilonacept at the 18MDM (*n* = 7), five (71%) had a pericarditis recurrence within 1–4 months of rilonacept suspension, despite all having had none/trace LGE (*n* = 7) and negative T2-STIR (*n* = 7) at the 18MDM and two having received prophylactic colchicine.

**Conclusion:**

Continued clinical improvement during prolonged rilonacept treatment corresponded with improvement on CMR, including reduced pericardial thickness, resolution of pericardial oedema, and resolution of LGE. However, none/trace LGE at 18MDM while on treatment did not predict absence of pericarditis recurrence upon subsequent rilonacept suspension in this size-limited subgroup.

## Graphical Abstract

**Graphical Abstract:**
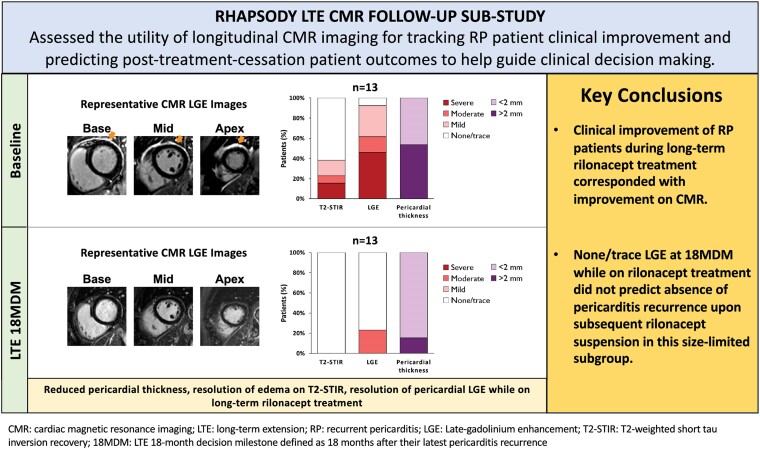

## Introduction

In the USA, the annualized prevalence of pericarditis is ∼ 160 000; of these, up to 40 000 have recurrent disease, and ∼14 000 experience multiple recurrences.^1–4^ Several studies have demonstrated similar results in Europe.^5,6^ Thus, recurrent pericarditis is an auto-inflammatory disease which can be chronic, with a median duration of at least 3 years, often requiring prolonged treatment.^5,7^

Many patients who experience multiple pericarditis recurrences demonstrate inadequate response to conventional therapy.^8^ Management typically includes activity restriction and medications such as non-steroidal anti-inflammatory drugs and colchicine, while those with difficult-to-treat disease can often achieve symptom control and remission with systemic corticosteroids; however, this approach increases the risk of other long-term adverse outcomes.^5,9^ Recent evidence has demonstrated efficacy in treating recurrent pericarditis by inhibiting the interleukin (IL)-1 pathway.^10,11^ In contrast to conventional therapy, targeted immunomodulation of the IL-1 pathway arrests the chronic auto-inflammatory state driving pericarditis recurrences and results in resolution of active flares and prevention of subsequent flares. In the Phase 3 trial, RHAPSODY, the IL-1α and IL-1β cytokine trap rilonacept effectively resolved active pericarditis recurrences and reduced the overall risk of recurrence during long-term treatment.^12^ Notably, patients who continued rilonacept treatment beyond 18 months experienced a 98% reduction in recurrence risk compared with those who discontinued rilonacept at that time (hazard ratio: 0.02; *P* < 0.0001).^13^

Despite encouraging therapeutic advances based on the evidence that recurrent pericarditis is mediated by the IL-1 pathway, substantial knowledge gaps remain about clinical management of the disease. For example, while it is understood at the population level that recurrent pericarditis is chronic, often lasting for years, the treatment duration for the individual patient is uncertain. Additional knowledge gaps include how to effectively monitor treatment response and how to predict future recurrences off-treatment, information which could help reduce anxiety and improve the quality of life for patients.^5,14,15^

Cardiac magnetic resonance (CMR) imaging with assessment of late gadolinium enhancement (LGE) can be used to assess pericardial inflammation and vascularity with high sensitivity.^16^ During RHAPSODY, CMR imaging revealed an association between higher pericardial LGE at baseline and shorter time to pericarditis recurrence for patients withdrawing from a 12-week rilonacept treatment run-in.^16^ This finding, when combined with observations from similar studies,^17–20^ supports the potential value for longitudinal assessment by serial CMR in evaluating clinical improvement, predicting patient outcomes, and informing treatment decisions for patients with recurrent pericarditis. To investigate this further, we performed long-term, serial CMR assessments while monitoring for recurrences inpatients who reached an 18-month decision milestone (18MDM) in the RHAPSODY long-term extension trial.

## Methods

### Study design

RHAPSODY was a Phase 3, double-blind, placebo-controlled, randomized-withdrawal trial that enrolled 86 patients with a history of two or more pericarditis recurrences and an active recurrence. The design of the primary study phase and long-term extension has previously been described.^13,21^ Briefly, eligible paediatric and adult patients received rilonacept while weaning from background pericarditis medications during a 12-week run-in period, after which those who met pre-specified clinical response criteria were randomized to weekly rilonacept or placebo in an event-driven randomized-withdrawal period (see Supplementary data online, *Figure S1*). Clinically stable patients who completed the randomized-withdrawal period were eligible to continue and receive weekly rilonacept in an open-label, long-term extension, while those who had achieved clinical response criteria but were still in the run-in when the randomized-withdrawal period ended could enter the long-term extension directly. Concomitant oral pericarditis medications (except for corticosteroids) could be resumed during the long-term extension. Patient participation ended after 24 months of treatment, upon treatment discontinuation, or at commercial launch of rilonacept in the US (US patients only). The long-term extension included a decision milestone 18 months after each patient's most recent pericarditis recurrence (initial qualifying event or flare during the randomized-withdrawal period) when the investigator decided through using shared decision-making and clinical judgement, implemented whether if the patient would continue rilonacept, suspend rilonacept but remain in the study for off-treatment observation, or discontinue the study without further observation. Pericardial CMR was performed at selected study sites at baseline run-in (up to 7 days prior to rilonacept initiation), and at the 18MDM. Patients were also monitored for investigator-assessed pericarditis recurrence, and all patients were evaluated at a 6-week safety follow-up assessment at the end of the trial.

RHAPSODY was conducted in accordance with the principles of the Declaration of Helsinki, the Good Clinical Practice Guidelines of the International Council for Harmonization of Technical Requirements for Pharmaceuticals for Human Use, and all relevant regulations. The protocol was approved by the respective institutional review boards or independent ethics committees at each study site. All patients provided written informed consent.

### Procedures and assessments

CMR assessments were performed according to standard protocol using a Philips Achieva™ 1.5T, Philips Ingenia® 3T, Siemens MAGNETOM Aera® 1.5T, Siemens MAGNETOM Vida® 3T, GE Optima™ MR450w 1.5T, or GE Signa™ HDxt 1.5T scanner. For LGE analyses, patients received gadolinium-based contrast agent, ∼10–20 min after which images were obtained in long- and short-axis orientations using magnitude images and a phase-sensitive inversion recovery technique with an inversion time selected for optimal nulling of the myocardium. Image analysis was performed using CVI42® (Circle Cardiovascular Imaging Inc. Calgary, Canada) and reviewed by imaging cardiologists with level III expertise in accordance with the Society of Cardiovascular Magnetic Resonance, and who were blinded to patient and clinical data (D.H.K. and C.L.J.). Pericardial thickness was assessed on black blood imaging, and pericardial oedema [T2-short tau inversion recovery (STIR) fat saturation] and LGE were graded using the pre-specified criteria summarized in *Table 1*.^16^ At 18MDM, improvement from baseline was defined as a lower grading (LGE, T2-STIR) and/or decrease in pericardial thickness measurement (<2 mm in absolute thickness was considered normal).

**Table 1:** Grading criteria for pericardial oedema and LGE

Grade	Definition
None	No signal detected
Trace	Increased signal limited to < 50% of cardiac circumference at least 1 of 3 ventricle levels (base/mid-cavity/apex)
Mild	Increased signal involving > 50% of cardiac circumference at 1 of 3 ventricle levels (base/mid-cavity/apex)
Moderate	Increased signal involving > 50% of cardiac circumference at 2 of 3 ventricle levels (base/mid-cavity/apex)
Severe	Increased signal in > 50% of cardiac circumference at all 3 ventricle levels (base/mid-cavity/apex)

LGE, late gadolinium enhancement.

During the conduct of the long-term extension, presence of pericarditis recurrence was investigator-assessed, which included evaluation of patient-reported pain, measurement of inflammatory markers, and need for treatment intensification. Measures of pain as assessed on numeric rating scale, C-reactive protein (CRP) concentration, electrocardiogram (ECG) assessments, pericardial effusion based on echocardiography, presence/absence of pericardial friction rub, Patient Global Impression of Pericarditis Severity, and Physician Global Assessment of Pericarditis Activity, were collected and examined in the context of RHAPSODY endpoint adjudication criteria for this *post hoc* review.^12^

### Statistical analysis

Given the small sample size, no formal statistical comparisons were performed for this analysis. All data are presented descriptively.

## Results

### Patient characteristics

**Figure 1:**
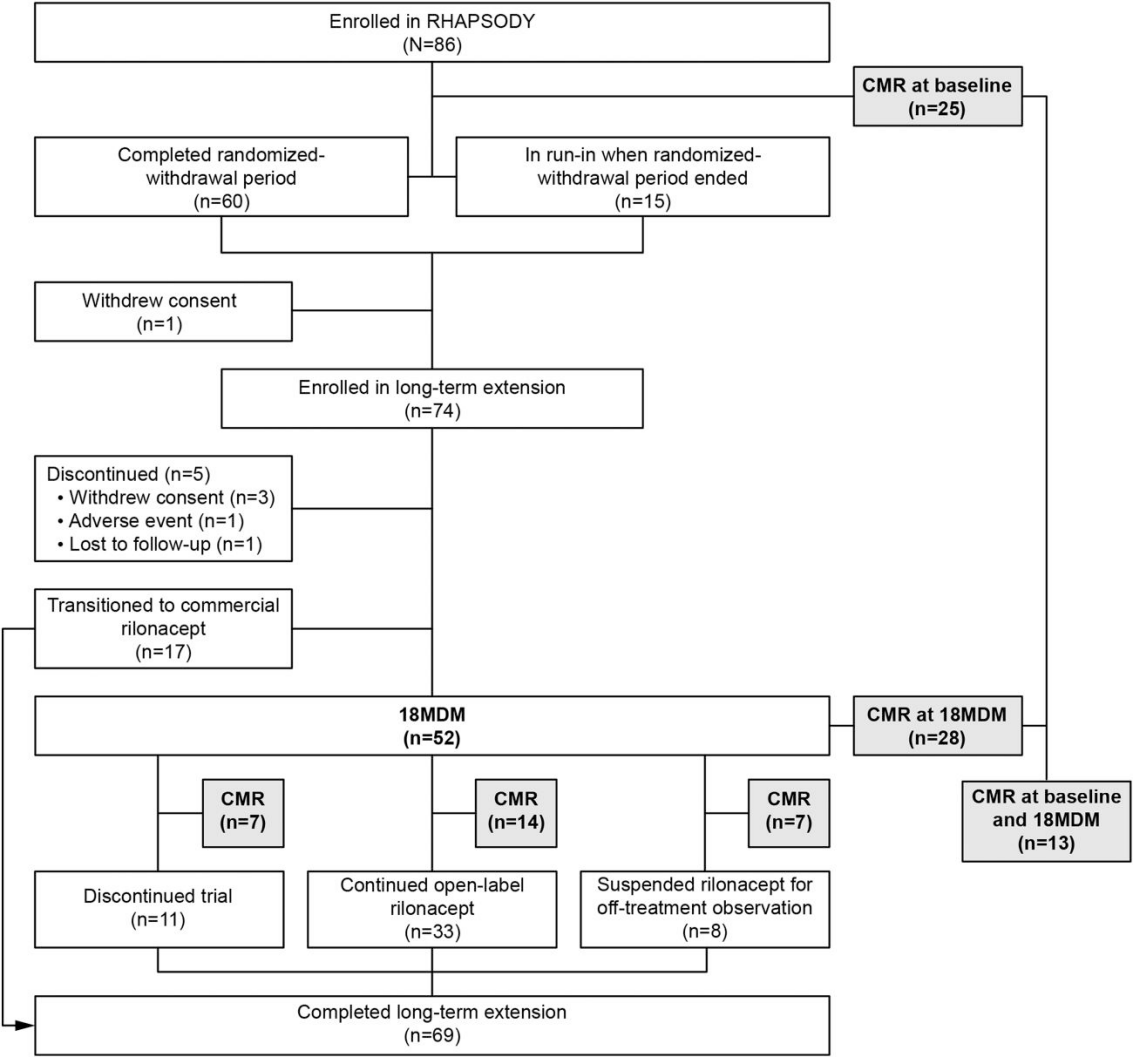
Patient disposition.

Of 86 patients who enrolled in RHAPSODY, 74 entered the open-label, long-term extension. A total of 52/74 (70.3%) patients reached the 18MDM, of whom 28/52 (53.8%) had CMR assessments at the 18MDM, and 13/52 (25.0%) had CMR assessed at both initial run-in (baseline) and 18MDM (*Figure 1*). Among the 28 patients with CMR at 18MDM, the mean [±standard deviation (SD)] age at enrolment was 43.1 (±15.6) years, 53.6% were female, median disease duration was 1.6 years (range: 0.4–23.8), and the predominant underlying aetiology was ‘idiopathic’ (92.9%) (*Table 2*).

**Table 2:** Patient baseline characteristics

	CMR assessments	
Characteristic	18MDM (*n* = 28)	Baseline + 18MDM (*n* = 13)
Age, years, mean ± SD	43.1 ± 15.6	45.4 ± 11.4
Sex, *n* (%)		
Male	13 (46.4)	4 (30.8)
Female	15 (53.6)	9 (69.2)
Race, *n* (%)		
White	27 (96.4)	12 (92.3)
Black or African American	1 (3.6)	1 (7.7)
Other	0	0
Recurrent pericarditis type, *n* (%)		
Idiopathic	26 (92.9)	12 (92.3)
Post-pericardiotomy syndrome	2 (7.1)	1 (7.7)
Dressler syndrome	0	0
Pericarditis treatment at baseline, *n* (%)	26 (92.9)	12 (92.3)
Corticosteroid	15 (53.6)	5 (38.5)
Other	23 (82.1)	12 (92.3)
Disease duration, years, median (range)	1.6 (0.4–23.8)	1.8 (0.7–23.8)
Total pericarditis episodes, including index/qualifying episodes, mean ± SD	4.9 ± 1.7	4.7 ± 1.3
Annualized incidence of pericarditis episodes, mean ± SD	3.3 ± 2.7	3.0 ± 2.7
CRP level (qualifying episode), mg/dL, mean ± SD	5.8 ± 7.1	3.8 ± 1.7

### Change in CMR from baseline to 18MDM

**Figure 2:**
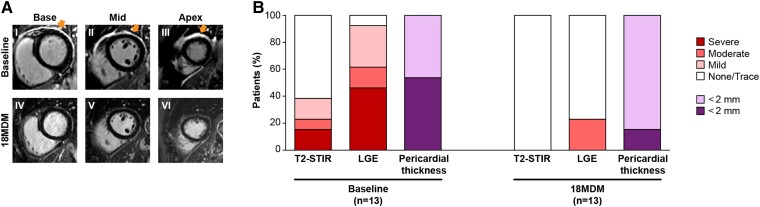
(*A*) Representative LGE imaging sequences performed at baseline and at the 18MDM for a patient that suspended rilonacept treatment at 18MDM and had a recurrence 4 months later. Slices at the base (Panels I and IV), mid (Panels II and V), and apex (Panels III and VI) of the left ventricle are included. Baseline images (Panels I–III) show severe pericardial LGE (arrows), while images at the 18MDM (Panels IV–VI) show resolution of LGE. (*B*) Change from baseline to 18MDM for all patients with data at both baseline and 18MDM.

Of the patients who had CMR assessments at both baseline and 18MDM, 12/13 (92.3%) showed improvement in LGE, T2-STIR, and pericardial thickness. Representative LGE CMR images from a patient are shown in *Figure 2A*, and results for all patients are summarized in *Figure 2B* and *Table 3*. Representative T2-STIR images are shown in Supplementary data online, *Figure S2*. At baseline 5/13 (38.4%) patients had elevated T2-STIR, 12/13 (92.3%) had abnormal LGE, and 7/13 (53.8%) had pericardial thickness >2 mm, whereas, at the 18MDM, 0/13 patients had elevated T2-STIR, 3/13 (23.1%) had at least some degree of elevated LGE, and 2/13 (15.4%) had pericardial thickness >2 mm.

**Table 3:** Summary of results for patients with CMR LGE data at baseline and 18MDM

Patient	Baseline disease duration (years)	18MDM assignment	Baseline CMR			18MDM CMR			Time from 18MDM to recurrence
			LGE	T2-STIR	Pericardial thickness (mm)	LGE	T2-STIR	Pericardial thickness (mm)	
1	1.7	Continue rilonacept	Severe	Severe	2.1	Moderate	Neg	1.4	No recurrence
2	1.3	Continue rilonacept	Severe	Mild	2.5	None	Neg	1.4	No recurrence
3	0.7	Continue rilonacept	Mild	Neg	5.4	None	Neg	2.7	No recurrence
4	3.3	Suspend for observation	Mild	Neg	1.2	None	Neg	0.9	2.8 months post-18MDM
5	3.2	Suspend for observation	Moderate	Neg	1.2	Trace	Neg	1.3	2.5 months post-18MDM
6	2.9	Suspend for observation	Severe	Neg	1.7	Trace	Neg	0.9	4.1 months post-18MDM
7	1.4	Suspend for observation	Mild	Neg	1.2	None	Neg	1.2	No recurrence
8	1.4	Suspend for observation	Severe	Neg	3.3	Trace	Neg	1.5	No recurrence
9	23.8	Discontinue trial	Severe	Mod	2.5	None	Neg	1.4	No recurrence
10	3.1	Discontinue trial	Trace	Neg	1.2	None	Neg	2.3	No recurrence
11	2.2	Discontinue trial	Moderate	Mild	1.6	Moderate	Neg	1.8	No recurrence
12	1.2	Discontinue trial	Mild	Neg	1.4	None	Neg	1.5	No recurrence
13	0.9	Discontinue trial	Severe	Severe	2.4	Moderate	Neg	1.3	No recurrence

18MDM, 18-month decision milestone; CMR, cardiac magnetic resonance; LGE, late gadolinium enhancement.

### CMR and clinical outcomes at/after 18MDM

Of the 28 patients who reached the 18MDM, 14 continued rilonacept, 7 suspended rilonacept for off-treatment observation, and 7 discontinued the study. All 28 patients had a CRP concentration <1 mg/dL, normal ECG, no pericardial rub, and no pericardial effusion at the 18MDM.

The 18MDM CMR assessment was performed on all 28 patients while still receiving open-label rilonacept. Of the 14 patients with CMRs who would continue rilonacept, 12/14 (85.7%) had none/trace LGE, and 2/14 (14.3%) had mild and moderate LGE at this time. None of the 14 patients experienced a pericarditis recurrence over the subsequent course of continued rilonacept treatment; however, 2/14 patients (14.3%) had a recurrence after treatment cessation recorded at the 6-week post-treatment safety follow-up visit. These two patients had received rilonacept treatment for a total of 25 and 28 months, respectively, and neither had demonstrable LGE at the 18MDM CMR assessment.

**Figure 3:**
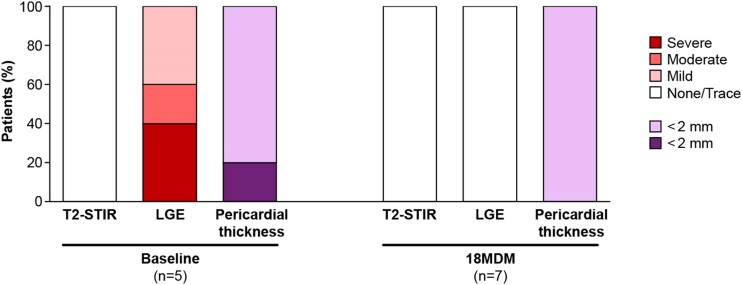
CMR results at baseline and 18MDM for patients who suspended rilonacept for off-treatment observation. Of 8 patients who suspended rilonacept for off-treatment observation at 18MDM, CMR was not assessed for 3 at baseline and 1 at 18MDM. All eight patients were negative for symptoms, CRP, ECG, echo/effusion, and exam/rub at 18MDM.

CMR results for the seven patients who suspended rilonacept for off-treatment observation (5 of whom also had results at baseline) are shown in *Figure 3*, with further details provided in Supplementary data online, *Figure S3*. At the 18MDM assessment, all seven patients had none/trace LGE, normal T2-STIR, and pericardial thickness <2 mm. During the off-treatment observation period, 5/7 (71.4%) patients had recurrences within 1–4 months after rilonacept suspension, including 2 patients who were receiving prophylactic colchicine during that time.

Of the seven patients who discontinued the study at the 18MDM, four (57.1%) had none/trace LGE, one (14.3%) had mild LGE, and two (28.6%) had moderate LGE at that time. There were no pericarditis recurrences after treatment cessation that were recorded at the 6-week post-treatment safety follow-up visit.

## Discussion

In this sub-study of the pivotal Phase 3 trial RHAPSODY, patients who received continuous rilonacept treatment for 18 months after their latest recurrence demonstrated reduced pericardial thickness, resolution of pericardial oedema on T2-STIR, and resolution of pericardial LGE on CMR imaging that corresponded with their continued clinical improvement. However, absence of LGE at 18MDM while on rilonacept treatment did not predict absence of pericarditis recurrence upon subsequent rilonacept suspension. Although these findings extend our previous work demonstrating a significant reduction in risk of pericarditis recurrence with continued, long-term rilonacept treatment,^12,13^ they emphasize that additional work must be done to elucidate whether resolution of pericardial LGE could inform treatment duration and the potential for persistent remission upon subsequent treatment suspension.^16,19^

This sub-study was intended to evaluate the potential utility of serial CMR data for guiding clinical decision-making inpatients with recurrent pericarditis. Results from a previous quantitative assessment suggested that a greater magnitude of pericardial LGE on CMR was associated with adverse clinical outcomes and a faster time to recurrence upon treatment cessation.^16,19^ In this sub-study, we observed improvements in pericardial LGE, T2-STIR, and pericardial thickness and a corresponding lower frequency of pericarditis recurrence among patients who received long-term rilonacept, supporting the use of CMR for following and documenting the effective management of recurrent pericarditis during treatment. However, our data, within the limitations of small numbers, did not reveal any obvious association between the absence of LGE by CMR at 18MDM and a protection from future recurrence upon subsequent rilonacept treatment suspension. Specifically, despite all patients in the off-treatment observation group having had normal T2-STIR and negative LGE at the 18MDM, five of the seven had a recurrence during the post-suspension observation period. Additionally, among patients who continued rilonacept on-study (*n* = 14 with CMR data), the two patients who had a recurrence post-treatment cessation recorded during the safety follow-up in fact had negative LGE at the 18MDM (see *Figure 2*), whereas the two patients who had some degree of persistent LGE at the 18MDM and did not continue onto commercial rilonacept after the study (after 7.1 and 4.5 months, respectively, of additional rilonacept treatment after 18MDM) nevertheless had no recurrence during the 6-week post-treatment follow-up. Finally, none of the patients in the discontinuation group (*n* = 7) with CMR data had a recurrence during the 6-week safety follow-up, despite three of seven having elevated LGE (one mild and two moderate) at the 18MDM. However, these results need to be interpreted with caution, as the patients who suspended rilonacept were observed off-treatment for an extended period of time, up to an additional 6 months, whereas those in the other groups were observed for only up to 6 weeks after discontinuation as part of the safety follow-up. Median time to recurrence after rilonacept cessation was previously reported in the RHAPSODY randomized-withdrawal period as 8.6 and 11.8 weeks in the LGE.^13^ Since the recurrences in the off-treatment observation group occurred 1–4 months after treatment discontinuation, it is possible that more recurrences might have been detected in the other groups had there been a longer follow-up.

The severity of pericardial LGE at recurrent pericarditis (RP) presentation is an important diagnostic and prognostic tool to help identify patients who require immediate and potentially prolonged IL-1 pathway inhibition. Tracking LGE improvement while on IL-1 pathway inhibition confirms both the initial RP diagnosis as well as treatment response. Although monitoring resolution of LGE is valuable in highlighting absence of neo vessels to indicate control of the underlying active auto-inflammation, absence of LGE while on RP treatment is not predictive of whether the underlying disease is still present or whether the auto-inflammation could become reactivated once suppressive therapy has been withdrawn or suspended. Larger prospective studies will be required to further elucidate the value of CMR in particular as a potential biomarker for predicting patient outcomes after treatment cessation and guiding treatment decisions, such as the required duration of treatment for recurrent pericarditis, and how treatment patterns may influence associated clinical outcomes.

### Limitations

Despite the value provided by these results, there were several limitations that need to be considered. First, this study was limited by a small number of patients with available CMR data. Secondly, there was no stipulation for specific CMR results or other requirements to inform patient management decision at the 18MDM. Thirdly, pericarditis recurrence events during the long-term extension were based on investigator assessment and were not externally adjudicated, although the clinical data were collected and examined in *post hoc* event review. Finally, as previously mentioned, for patients who discontinued the study at 18MDM and those who continued rilonacept until the end of the study, recurrence events occurring after the end of the 6-week safety follow-up period would have been outside the observation period of the trial, so the observed recurrences for these patients may have been underestimated.

## Conclusion

In conclusion, in this CMR and clinical analysis of patients treated with rilonacept for recurrent pericarditis, continued clinical improvement during prolonged rilonacept treatment corresponded with improvement on CMR, including reduced pericardial thickness, resolution of pericardial oedema on T2-STIR, and resolution of LGE. These findings suggest the clinical usefulness of CMR to diagnose and monitor pericardial inflammation during clinical follow-up. Nevertheless, none/trace LGE at 18MDM while on rilonacept treatment did not predict absence of pericarditis recurrence upon subsequent rilonacept suspension in this size-limited subgroup, suggesting the importance of a prolonged therapy in this subset of patients. Larger prospective studies examining CMR parameters in guiding decisions on treatment duration and informing associated clinical outcomes are warranted.

## Supplementary Material

jeae200_Supplementary_Data

## Data Availability

Data are available in a public, open access repository. Data are available upon reasonable request. De-identified participant data will be available, 1 year after study completion, upon reasonable written request to researchers whose proposed use of the data has been approved. Requests may be directed to J.F.P., (ORCID ID: 0000-0001-7622-8851). Data will not be provided to requesters with potential or actual conflicts of interest, including individuals requesting access for commercial, competitive, or legal purposes. Data access may be precluded for studies in which clinical data were subject to legal, contractual, or consent provisions that prohibit transfer to third parties. All those receiving access to data will be required to enter into a Data Use Agreement, which shall contain terms and conditions that are customary for similar agreements and similar companies in the industry. Data available to the public, including the study protocol, can be found at: (data set) Klein AL, Imazio M, Cremer P, *et al.* Phase 3 study of interleukin-1 trap rilonacept in recurrent pericarditis. *N Engl J Med* 2021;**384**:31–41. DOI: 10.1056/NEJMoa2027892.
