# Supplementary material for: Longitudinal cardiac magnetic resonance imaging following clinical response to rilonacept and prior to recurrence upon treatment suspension: a RHAPSODY subgroup analysis
Source: Eur Heart J Cardiovasc Imaging. 2024 Aug 22;26(1):72–9. doi: 10.1093/ehjci/jeae200 (PMC11687114; doi:10.1093/ehjci/jeae200)
Supplement: jeae200_Supplementary_Data [file jeae200_supplementary_data.docx]

# Longitudinal Cardiac Magnetic Resonance Imaging Following Clinical Response to Rilonacept and Prior To Recurrence Upon Treatment Suspension: a RHAPSODY Subgroup Analysis

# Supplementary Material

| **Table S1. Patient status** | | | | | | |
| --- | --- | --- | --- | --- | --- | --- |
| **Patient** | **Baseline disease  duration (y)** | **18MDM assignment** | **CMR LGE Result** | | | **Time from 18MDM**  **to post-18MDM recurrence** |
|  |  |  | **Baseline** | **18MDM** | **EOT** |  |
| 14 | 11.2 | Continue rilonacept | - | None | None | 10.4 months* |
| 15 | 4.6 | Continue rilonacept | - | None | - | No recurrence |
| 16 | 3.0 | Continue rilonacept | - | None | - | No recurrence |
| 17 | 2.5 | Continue rilonacept | - | NM | - | No recurrence |
| 18 | 2.2 | Continue rilonacept | - | None | - | No recurrence |
| 19 | 1.8 | Continue rilonacept | - | None | Mild | 8.4 months* |
| 1 | 1.7 | Continue rilonacept | Severe | Moderate | - | No recurrence |
| 20 | 1.5 | Continue rilonacept | - | None | Trace | No recurrence |
| 21 | 1.5 | Continue rilonacept | - | None | - | No recurrence |
| 22 | 1.4 | Continue rilonacept | - | None | - | No recurrence |
| 2 | 1.3 | Continue rilonacept | Severe | None | - | No recurrence |
| 23 | 0.8 | Continue rilonacept | - | Mild | - | No recurrence |
| 3 | 0.7 | Continue rilonacept | Mild | None | - | No recurrence |
| 24 | 0.4 | Continue rilonacept | - | None | - | No recurrence |
| 4 | 3.3 | Suspend for off-treatment observation | Mild | None | Moderate | 2.8 months |
| 5 | 3.2 | Suspend for off-treatment observation | Moderate | Trace | - | 2.5 months |
| 6 | 2.9 | Suspend for off-treatment observation | Severe | Trace | Trace | 4.1 months |
| 25 | 1.5 | Suspend for off-treatment observation | - | None | - | 2.8 months |
| 7 | 1.4 | Suspend for off-treatment observation | Mild | None | - | No recurrence |
| 8 | 1.4 | Suspend for off-treatment observation | Severe | Trace | - | No recurrence |
| 26 | 0.5 | Suspend for off-treatment observation | - | None | - | 0.8 months |
| 9 | 23.8 | Discontinued study | Severe | None | - | No recurrence |
| 10 | 3.1 | Discontinued study | Trace | None | - | No recurrence |
| 11 | 2.2 | Discontinued study | Moderate | Moderate | - | No recurrence |
| 27 | 2.0 | Discontinued study | - | Trace | - | No recurrence |
| 28 | 1.5 | Discontinued study | - | Mild | - | No recurrence |
| 12 | 1.2 | Discontinued study | Mild | None | - | No recurrence |
| 13 | 0.9 | Discontinued study | Severe | Moderate | - | No recurrence |
| *Patient stopped rilonacept treatment at the end of study and the recurrence occurred during the post-study 6-week safety follow up 1.4 months after rilonacept cessation.  18MDM, 18-month decision milestone; CMR, cardiac magnetic resonance; EOT, end of treatment; NM, not measurable | | | | | | |

**Figure S1.** RHAPSODY study design, including patients in the CMR sub-study.


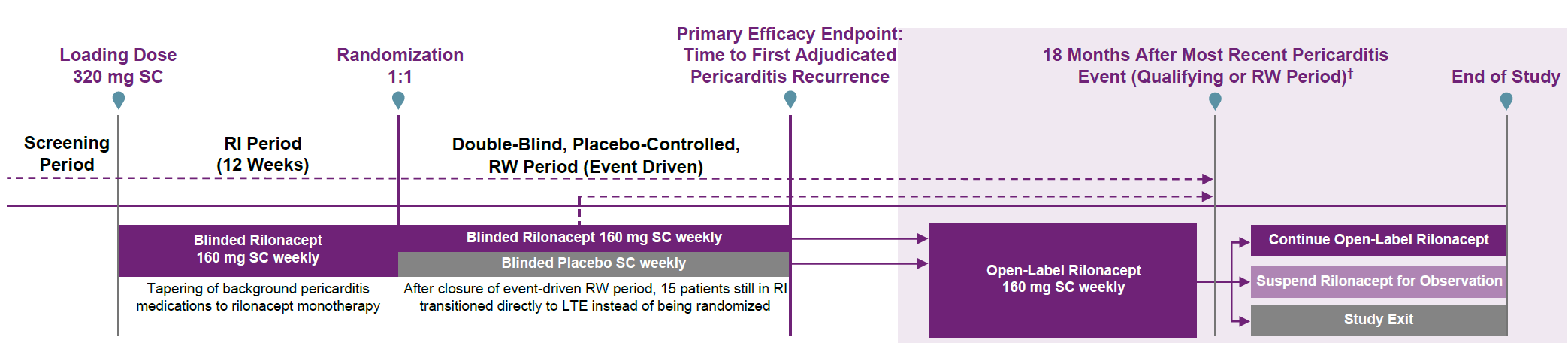


Assessments at 18MDM: CMR, pain by numeric rating scale, C-reactive protein; electrocardiogram, effusion (echocardiogram), pericardial friction rub, Patient Global Impression of Pericarditis Severity, Physician Global Assessment of Pericarditis Activity.

**Figure S2.** Representative T2-STIR (fat saturation) images with grading from a patient showing improvement from baseline to 18MDM (arrows indicate pericardial edema).

**
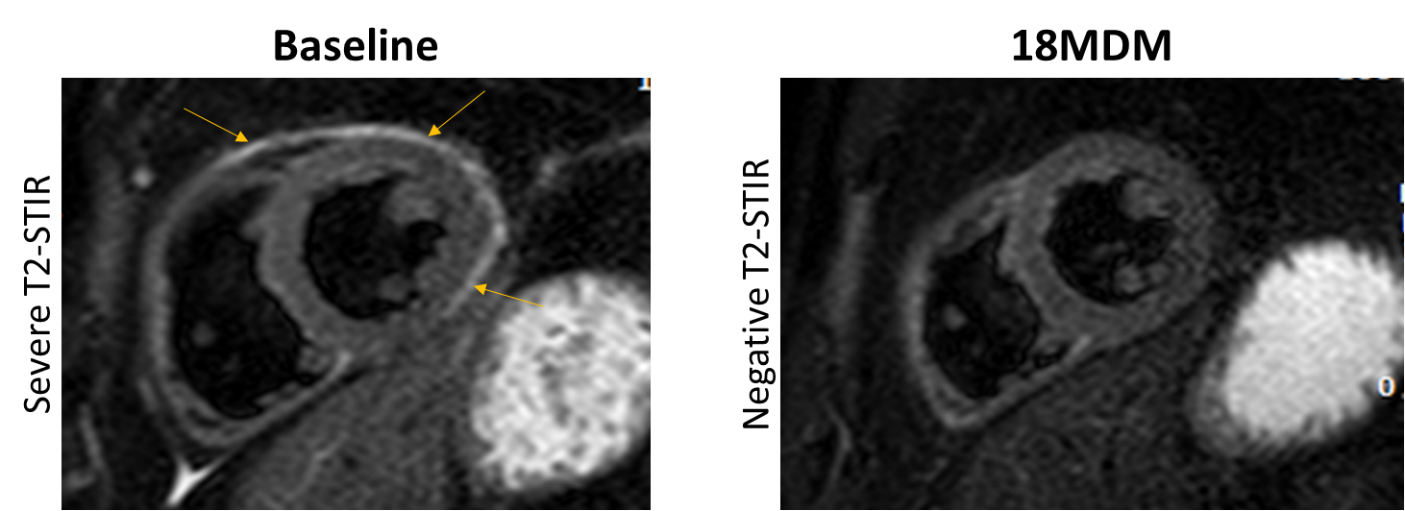
**

**Figure S3.** Diagram showing change in CMR LGE severity from baseline to 18MDM for patients with LGE data at baseline and 18MDM^a^

**
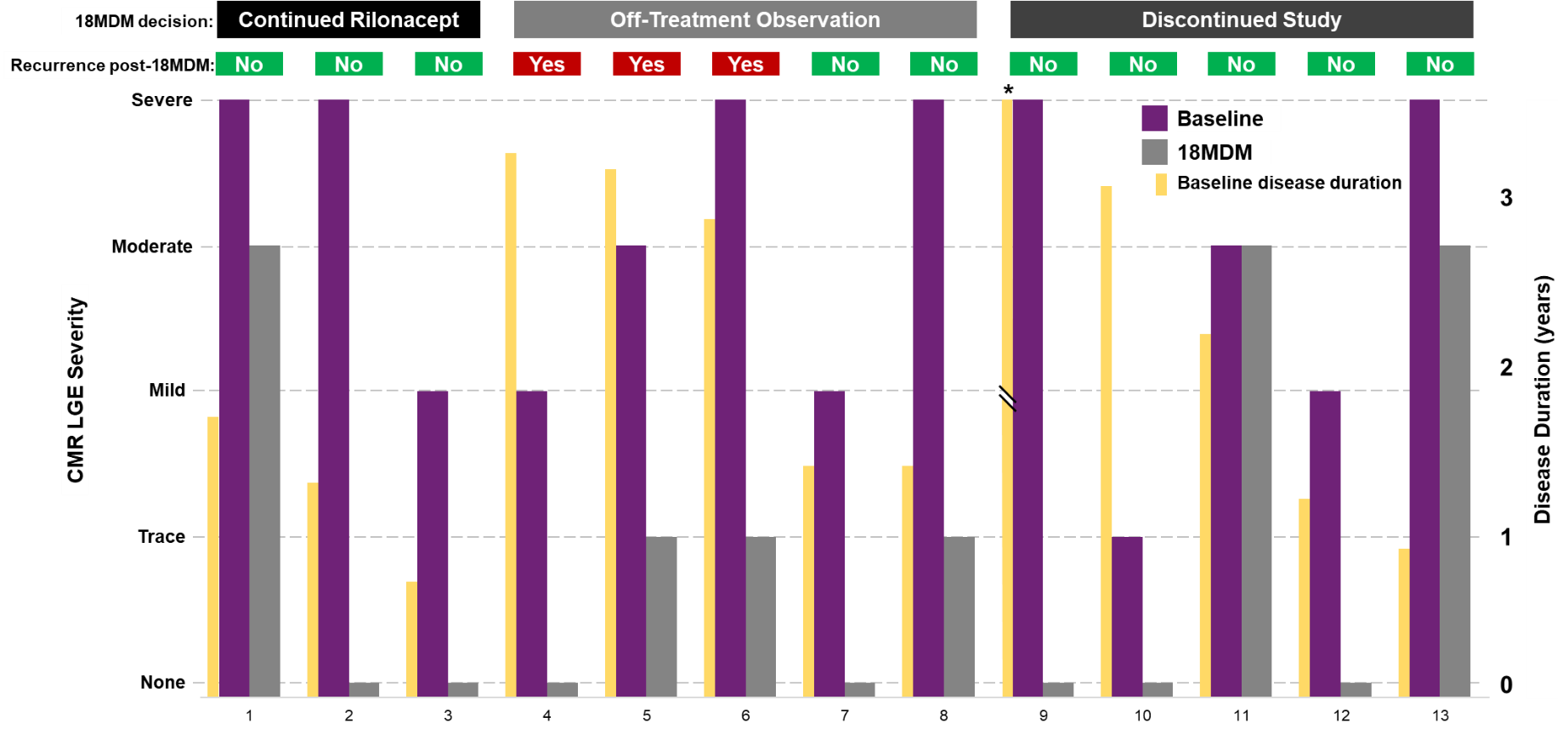
**

^a^Patients who continued rilonacept until end of study or discontinued the study at the 18MDM were followed for a maximum of 6 weeks (safety follow-up) after cessation of rilonacept at end of study.
*Baseline disease duration: 23.8 years.
